# Persistence of Mental Health Deterioration Among People Living Alone During the COVID-19 Pandemic: A Periodically-repeated Longitudinal Study

**DOI:** 10.2188/jea.JE20210397

**Published:** 2022-07-05

**Authors:** Hiroyuki Kikuchi, Masaki Machida, Itaru Nakamura, Reiko Saito, Yuko Odagiri, Noritoshi Fukushima, Tomoko Takamiya, Shiho Amagasa, Keisuke Fukui, Takako Kojima, Hidehiro Watanabe, Shigeru Inoue

**Affiliations:** 1 Department of Preventive Medicine and Public Health, Tokyo Medical University, Tokyo, Japan; 2 Department of Infection Prevention and Control, Tokyo Medical University Hospital, Tokyo, Japan; 3 Division of International Health (Public Health), Graduate School of Medical and Dental Sciences, Niigata University, Niigata, Japan; 4 Department of International Medical Communications, Tokyo Medical University, Tokyo, Japan; 5 Graduate School of Advanced Science and Engineering, Hiroshima University, Hiroshima, Japan

**Keywords:** living arrangement, novel coronavirus, K6, psychological distress

## Abstract

**Background:**

This longitudinal study aimed to investigate how psychological distress levels changed from early to middle phases of the new coronavirus (COVID-19) pandemic depending on the living arrangements of individuals.

**Methods:**

An internet-based, longitudinal survey of 2,400 Japanese people was conducted every 5–6 weeks between February 2020 and January 2021. The presence of severe psychological distress (SPD) was measured using the Kessler’s psychological distress scale. Living arrangements were classified into two groups (ie, living alone or living with others). Mixed-effects logistic regression analysis was performed to assess whether changes in SPD status were different depending on living arrangements.

**Results:**

Of 2,400 respondents, 446 (18.5%) lived alone. Although the proportion of SPD in both individuals living alone and those living with others increased to the same extent in the early phase of the pandemic, the distress levels decreased after the early phase of the pandemic in the group living with others, compared with the group living alone, for which SPD remained high. The odds ratio (OR) of developing SPD in interaction term with survey phases tended to be higher among those who lived alone than those who lived with others in Phase 6 (OR 1.89; 95% confidence interval [CI], 0.99–3.64) and Phase 7 (OR 1.88; 95% CI, 0.97–3.63).

**Conclusion:**

During the COVID-19 pandemic, those living alone are persistently at a higher risk of SPD compared to those living with others. Effective countermeasures targeting those living alone, such as enhancing online communication or providing psychological therapies, are essential.

## INTRODUCTION

As of August 25, 2021, more than 400 million people have been infected by the novel coronavirus 2019 (COVID-19), and more than 2 million people have died worldwide.^1^ This new infectious disease pandemic deteriorates people’s mental health through fear and anxiety of infection, isolation, and death, as well as increased loneliness due to social isolation and other factors.^2^^,^^3^ It has also been reported that serious secondary damages, such as domestic violence, abuse, and suicide, are increasing as a result of deteriorating mental health.^2^^,^^4^^,^^5^ Thus, the World Health Organization (WHO) requires that countermeasures against COVID-19 include both infection preventive measures and mental health measures at the same time.^6^

The COVID-19 infection control measures focus on reducing face-to-face contacts in society, which in turn further reduces the social support provided from outside the family.^7^^,^^8^ Such measures have a negative impact on mental health, especially for those who live alone because they cannot receive adequate social support at home. Since March 2020, the government has repeatedly demanded that citizens limit their social activities. It can be hypothesized that the longer such restriction continues, the worse people’s mental health will become, especially for those who live alone.

Several longitudinal studies have investigated how mental health among people living alone has changed during the COVID-19 pandemic.^9^^,^^10^ However, the results of these studies have been inconsistent, as some studies report that mental health of those living alone have become worse than those living with others,^11^ while other studies report no association.^10^^,^^12^ Moreover, these previous studies were conducted only in the early phase of the COVID-19 pandemic. A recent Japanese study showed that the suicide rate declined substantially during the first wave of the COVID-19 pandemic (February to June 2020), but increased rapidly during the second outbreak (July to October 2020).^13^ This suggests that more attention should be given to psychological distress changes, not only in the early phase, but also in the later phases of the pandemic. In addition, another study reported that excess suicide death rate was observed among women but not men, possibly due to unstable employment status or increased susceptibility to violence,^5^ implying that the impact on psychological distress given by the COVID-19 pandemic may be different by gender. Therefore, this longitudinal study aimed to investigate how psychological distress levels changed during this pandemic depending on the living arrangement of individuals in Japan.

## METHODS

### Study sample and data collection

This was an online longitudinal study conducted among the Japanese population. The details of this study are only briefly addressed here, since the participant extraction method was described in detail in our previous study.^14^ The 8,156 participants who were approached to be part of this current study were those registered with My Voice Co., Ltd., an Internet research company. The participants resided in seven prefectures (ie, Ibaraki, Tochigi, Gunma, Saitama, Chiba, Kanagawa, and Tokyo), including the Tokyo Metropolitan area. In order to obtain responses from a total of 2,400 participants, 200 participants from each gender/age group (20s–70s) were selected. The study was conducted using the following procedure: 1) A questionnaire was uploaded onto a secured online platform; 2) the online survey company sent the URL of the questionnaire to its registered users; 3) respondents who received the URL accessed the online questionnaire, and responded voluntarily; and 4) responses were closed at the point where the set quotient had been met (ie, 200 respondents per each gender/age group). Survey respondents registered on the site were given paid points equivalent to 50 Japanese yen (JPY; approximately 0.5 United States dollars [USD]) as incentives for each completed survey.

### Survey dates and COVID-19 situation in Japan

**Figure 1.**
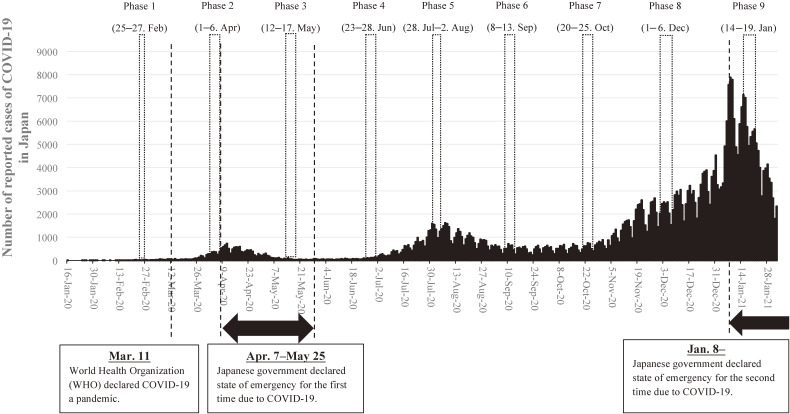
Date of surveys with COVID-19 epidemic curve in Japan

Survey dates with COVID-19 pandemic curve are shown in Figure 1. The baseline survey (Phase 1) was conducted from February 25–27, 2020, before the second wave of infection occurred in Japan. These dates correspond with the early phase of the COVID-19 outbreak in Japan. The number of COVID-19 cases in Japan, up to that point, consisted mainly of people returning from areas with an outbreak (eg, China) and those who had come into contact with such people. As such, there were not many infected people whose route of infection could not be traced. The total number of patients infected with COVID-19 up to February 25, the day before the survey, was 157 in Japan, with 1 death (note: this patient had been infected upon a cruise ship from China that docked at a Japanese port, and therefore was not a case in which COVID-19 was contracted domestically).^1^

The second (Phase 2: April 1–7) and third surveys (Phase 3: May 12–17) were conducted during the first wave of domestic infection of COVID-19. During these phases, the WHO had pronounced COVID-19 to be a pandemic (March 11). In response to increasing domestic infection, the Japanese government declared a state of emergency on April 7.^15^^,^^16^ Japanese citizens refrained from going out, and companies and schools widely continued to halt their operations. By May 11, the day before the Phase 3 research took place, there was a total of 16,014 infected patients and 657 deaths.

The fourth (Phase 4: Jun 23–28), fifth (Phase 5: July 28–Aug 2), and sixth survey (Phase 6: Sep 8–13) were conducted during the second wave of domestic infection of COVID-19. After the government declared a state of emergency, the number of patients in the country has dropped significantly to less than 50 per day, so the government lifted the emergency declaration on May 25. At this time, concerns about balancing economic measures and infection prevention measures became the subject for much debate. The Cabinet Office estimated that the gross domestic product growth rate for FY2020 would be −4.5% compared to the previous year. In response to this acute economic recession, the government provided a financial aid of 100,000 JPY (approximately 935 USD) to all citizens around June. Despite the number of daily cases reaching more than 1,000, however, the government avoided declaring a state of emergency due to its negative economic impact.

The eighth (Phase 8: December 1–6, 2020) and ninth (Phase 9: January 14–19, 2021) surveys were conducted during the third wave of domestic infection. In response to the rapid increase of infected people, the government declared a state of emergency for the second time. This declaration strongly urged restaurants to close at 8 pm; however, schools and other facilities were allowed to operate as usual (ie, relatively looser restrictions were imposed this time compared with the previous emergency declaration).

In this study, we used data from participants who responded at least one surveys.

### Measurements

#### Assessment of severe psychological distress

In both the baseline and follow-up surveys, the Kessler’s six-item psychological distress scale (K6) was used to measure severe psychological distress (SPD).^17^ Since the K6 measures psychological distress in the general population using six simple items, it is broadly used in epidemiological studies in assessing depression or suicide prevention.^18^ Each item measures the extent of general non-specific psychological distress using a 5-point response: 0 ‘*none of the time*’, 1 ‘*a little of the time*’, 2 ‘*some of the time*’, 3 ‘*most of the time*’, and 4 ‘*all of the time*’; thus, the total scores ranged from 0–24. The K6 was translated into Japanese, and a previous study conducted in 164 Japanese adults has proved its internal consistency in relation to reliability (Cronbach’s alpha: 0.849) and validity (100% sensitivity and 69.3% specificity for screening mood and anxiety disorder).^19^ This study used the established protocol to define a score of 13 or above as having SPD.^20^

#### Assessment of living arrangements

The Internet research company provided the number of family members living with the individual at the time of the Phase 1 survey. This study defined those who responded with “zero” as the ‘living alone’ group, and those who responded with “one or more” as the ‘living with others’ group.

#### Covariates

In the baseline survey, participants reported their gender, age, residential area (Northern Kanto area [Ibaraki, Tochigi, and Gunma Prefectures], Saitama Prefecture, Chiba Prefecture, Kanagawa Prefecture, Tokyo Metropolis), working status (working, not working), smoking status (smokers, ex-smokers, non-smokers), alcohol consumption (never, seldom [1–4 times/week], often [5–7 times/week], daily walking time (less than 30 mins, 30–59 mins, 60 mins), regular annual vaccination (yes, no), and past medical history (hypertension, diabetes, heart disease, stroke, respiratory disease, kidney disease, cancer). Current/past medical history categorized into three groups (none, 1, 2+) according to the total number of comorbidities in statistical analysis. In addition, the Internet research company provided categorized data of educational attainment (junior or high school graduate, junior college graduate, university graduate or above, others) and personal annual income. (<2 million JPY, [approx. 18,600 USD], 2–4 million JPY [18,600–37,200 USD], 4–6 million JPY [37,200–55,800 USD], and ≥6 million JPY [55,800 USD and over]).

### Statistical analysis

The mean K6 score for both living arrangements were compared using mixed-effect linear regression adjusted for gender, age, residential area (prefecture), working status, personal annual income, education, smoking status, alcohol consumption, walking time, comorbidities (none, one, two or more), and regular vaccination.

Thereafter, a mixed-effect logistic regression model was used to examine the correlation between living arrangement and developing SPD by nesting each participant.^21^ In this analysis, in order to seek the difference of likelihood for developing SPD, fixed effects were set for all individual factors, survey phases (ie, phase 1 to 9), individual K6 scores during Phase 1, and also for interaction term between living arrangement and phase. In order to account for differences between age groups, the above regression analysis was performed using data for the entire participant population, followed by separate data for gender, each age group (ie, 20–39 years, 40–64 years and ≥65 years) and working status. Stata version 15.0 (Stata Corp., College Station, TX, USA) was used for statistical analysis.

### Ethical approval

This study was approved by the Ethics Committee of Tokyo Medical University, Tokyo, Japan (No: T2019-0234). Informed consent was obtained from all respondents.

## RESULTS

Table 1 shows the characteristics of participants according to living arrangements. Of those, 958 (49.0%) were men, average age was 49.7 years, and standard deviation (SD) was 16.3 years. Regarding living arrangements, 446 (18.5%) lived alone. On average, participants responded to 8.07 (SD, 1.86) surveys out of nine surveys.

**Table 1.** Characteristics of study participants

	*n*	%	Living with others		Living alone		*P*
	
			*n*	%	*n*	%	
**Overall**	2,400		1,954		446		

Gender							0.046
Men	1,200	50.0%	958	49.0%	242	54.3%	
Women	1,200	50.0%	996	51.0%	204	45.7%	
Age, years							<0.001
20–39	800	33.3%	615	31.5%	185	41.5%	
40–64	1,019	42.5%	839	42.9%	180	40.4%	
≥65	581	24.2%	500	25.6%	81	18.2%	
Working status							<0.001
No	885	36.9%	780	39.9%	105	23.5%	
Yes	1,515	63.1%	1,174	60.1%	341	76.5%	
Residential area							<0.001
Northern Kanto (Ibaraki, Tochigi, Gunma Prefectures)	221	9.2%	193	9.9%	28	6.3%	
Saitama Prefecture	385	16.0%	326	16.7%	59	13.2%	
Chiba Prefecture	339	14.1%	291	14.9%	48	10.8%	
Tokyo Metropolis	922	38.4%	696	35.6%	226	50.7%	
Kanagawa Prefecture	533	22.2%	448	22.9%	85	19.1%	
Education, years							<0.001
Junior or high school graduate (≤12 years)	559	23.3%	462	23.6%	97	21.7%	
Junior college graduate (13–15 years)	487	20.3%	424	21.7%	63	14.1%	
University graduate or above (≥16 years)	1,258	52.4%	978	50.1%	280	62.8%	
Other	96	4.0%	90	4.6%	6	1.3%	
Annual income, Japanese yen							<0.001
<2 million	1,044	43.5%	918	47.0%	126	28.3%	
2 million–<4 million	608	25.3%	440	22.5%	168	37.7%	
4 million–<6 million	353	14.7%	253	12.9%	100	22.4%	
≥6 million	327	13.6%	275	14.1%	52	11.7%	
Smoking status							0.262
Smoker	340	14.2%	266	13.6%	74	16.6%	
Ex-smoker	344	14.3%	283	14.5%	61	13.7%	
Non-smoker	1,716	71.5%	1,405	71.9%	311	69.7%	
Alcohol consumption							0.035
None	1,020	42.5%	832	42.6%	188	42.2%	
Seldom (1–4 days/week)	872	36.3%	691	35.4%	181	40.6%	
Often (5–7 days/week)	508	21.2%	431	22.1%	77	17.3%	
Walking time, mins/day							0.809
<30	1,207	50.3%	984	50.4%	223	50.0%	
30–59	792	33.0%	648	33.2%	144	32.3%	
≥60	401	16.7%	322	16.5%	79	17.7%	
Regular vaccinations							<0.001
No	1,315	54.8%	1,037	53.1%	278	62.3%	
Yes	1,085	45.2%	917	46.9%	168	37.7%	
Comorbidities							
Hypertension	453	18.9%	374	19.1%	79	17.7%	0.487
Diabetes	135	5.6%	112	5.7%	23	5.2%	0.634
Heart disease	68	2.8%	53	2.7%	15	3.4%	0.455
Stroke	25	1.0%	19	1.0%	6	1.3%	0.883
Respiratory disease	104	4.3%	86	4.4%	18	4.0%	0.732
Kidney disease	14	0.6%	12	0.6%	2	0.4%	0.678
Cancer	51	2.1%	47	2.4%	4	0.9%	0.046

*P*-value was calculated by chi-square test.

**Figure 2.**
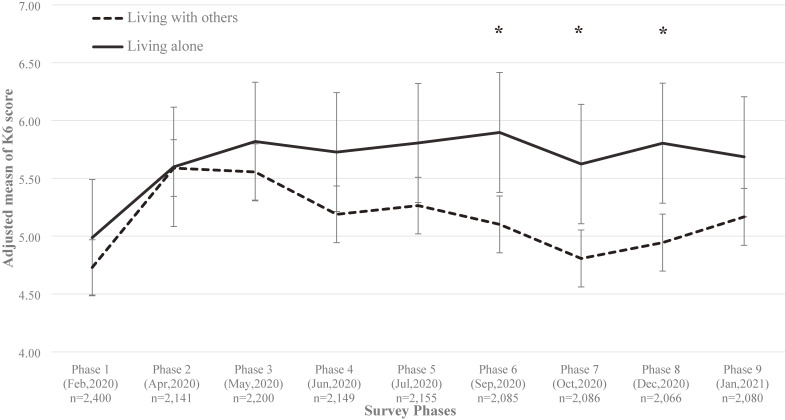
Adjusted means of K6 score according to living arrangements: mixed-effect linear regression results. K6, Kessler’s six-item psychological distress (The higher score indicates unfavourable distress state). ^*^ indicate statistically significant results in 5% significance levels, respectively. All mean values were adjusted by gender, age, residential area (prefecture), working status, personal annual income, education, smoking status, alcohol consumption, walking time, number of comorbidities (none, 1, 2+), and regular vaccination. Error bar indicate 95% confidence intervals.

Figure 2 shows the trajectory of the adjusted mean of K6 scores for each survey. According to the K6 score, psychological distress of both the ‘living alone’ and ‘living with others’ groups increased to the same extent in the early phase of the COVID-19 pandemic (ie, Phases 1–3). However, from Phase 4, psychological distress gradually improved in the ‘living with others’ group, while the ‘living alone’ group remained high. Mixed-effect linear regression showed that there were significant differences of K6 scores between both groups at phases 6–8 (*P* < 0.05).

Table 2 shows the number of participants and proportions of SPD by survey phases. In the pre-pandemic phase (Phase 1), 228 participants (9.5%) showed SPD. Among those who live with others, the highest proportion of SPD was observed at Phase 2, whereas the highest proportion was observed at phase 6 among those who live alone. Throughout the survey, men who live alone and the younger age group showed relatively higher proportion of SPD.

**Table 2.** The number of participants and proportions of SPD by survey phases

Survey phase			Phase 1 (Feb,2020)	Phase 2 (Apr,2020)	Phase 3 (May,2020)	Phase 4 (Jun,2020)	Phase 5 (Jul,2020)	Phase 6 (Sep,2020)	Phase 7 (Oct,2020)	Phase 8 (Dec,2020)	Phase 9 (Jan,2021)
Number of participants			2,400	2,141	2,200	2,149	2,155	2,085	2,086	2,066	2,080

Overall	Living with others	*n*	1,954	1,745	1,790	1,744	1,754	1,698	1,690	1,680	1,694
		Prop of SPD (%)	9.2%	11.5%	10.3%	8.9%	10.9%	9.6%	9.3%	9.3%	9.3%

	Living alone	*n*	446	396	410	405	401	387	396	386	386
		Prop of SPD (%)	11.0%	13.1%	11.7%	12.8%	15.2%	15.8%	14.4%	13.0%	13.7%

Men	Living with others	*n*	958	866	887	871	871	835	837	832	839
		Prop of SPD (%)	9.0%	11.3%	9.2%	8.7%	10.0%	9.2%	9.0%	9.3%	8.6%

	Living alone	*n*	242	221	224	225	223	211	218	214	218
		Prop of SPD (%)	13.2%	14.5%	14.3%	14.2%	17.5%	16.6%	14.7%	13.6%	14.7%

Women	Living with others	*n*	996	879	903	873	883	863	853	848	855
		Prop of SPD (%)	9.3%	11.6%	11.4%	9.0%	11.8%	10.0%	9.7%	9.3%	10.1%

	Living alone	*n*	204	175	186	180	178	176	178	172	168
		Prop of SPD (%)	8.3%	11.4%	8.6%	11.1%	12.4%	14.8%	14.0%	12.2%	12.5%

Younger age	Living with others	*n*	615	552	547	521	529	501	496	498	493
		Prop of SPD (%)	15.4%	19.4%	18.6%	17.3%	19.1%	19.4%	17.1%	16.9%	18.1%

	Living alone	*n*	185	157	163	160	159	152	156	148	150
		Prop of SPD (%)	17.3%	16.6%	11.7%	15.0%	18.9%	21.1%	17.9%	14.2%	16.7%

Middle age	Living with others	*n*	839	751	775	763	769	751	746	745	746
		Prop of SPD (%)	8.2%	9.9%	8.9%	7.6%	10.1%	7.3%	8.3%	8.2%	7.9%

	Living alone	*n*	180	167	172	170	169	164	165	168	164
		Prop of SPD (%)	7.8%	13.2%	14.0%	15.3%	15.4%	15.9%	15.8%	15.5%	14.6%

Older age	Living with others	*n*	500	442	468	460	456	446	448	437	455
		Prop of SPD (%)	3.0%	4.3%	3.0%	1.5%	2.6%	2.5%	2.5%	2.5%	2.2%

	Living alone	*n*	81	72	75	75	73	71	75	70	72
		Prop of SPD (%)	3.7%	5.6%	6.7%	2.7%	6.8%	4.2%	4.0%	4.3%	5.6%

SPD, severe psychological distress defined a score of 13 or above in Kessler’s six-item psychological distress scale.

Table 3 shows adjusted odds ratios (ORs) of developing SPD by living arrangement and survey phases. Overall, the likelihood of developing SPD was higher in Phase 2 (OR 1.53; 95% confidence interval [CI], 1.12–2.09), Phase 3 (OR 1.38; 95% CI, 1.01–1.87), and Phase 5 (OR 1.47; 95% CI, 1.08–2.01) compared with the pre-pandemic phase (Phase 1). This implies that a citizen’s mental health is more likely to deteriorate during the first 4 months of the pandemic. Regarding living arrangement, the association was non-significant overall (OR 1.19; 95% CI, 0.68–2.05), whereas the odds of developing SPD in interaction term with survey phases tended to be higher among those who lived alone than those who lived with others in Phase 6 (OR 1.89; 95% CI, 0.99–3.64) and Phase 7 (OR 1.88; 95% CI, 0.97–3.63). This indicates that during the first 4 months of the pandemic, the mental health of all citizens deteriorated regardless of whether they lived alone or not. Subsequently, psychological distress among those who lived with others gradually improved, while distress among those who lived alone remained severe. In the stratified analysis of gender, age group and working status, this tendency was particularly pronounced among women, middle-aged participants, and workers.

**Table 3.** Adjusted odds ratios in developing severe psychological distress according to living arrangement: mixed-effect logistic regression results

	Overall				Stratified by gender								Stratified by working status								Stratified by age											
		
					Men				Women				Not-working				Working				Younger age (20–39)				Middle age (40–64)				Older age (≥65)			
							
	*n*	OR	95% CI	*P*	*n*	OR	95% CI	*P*	*n*	OR	95% CI	*P*	*n*	OR	95% CI	*P*	*n*	OR	95% CI	*P*	*n*	OR	95% CI	*P*	*n*	OR	95% CI	*P*	*n*	OR	95% CI	*P*
Living arrangement																																
Living with others	1,954	1.00			958	1.00			996	1.00			780	1.00			1,174	1.00			615	1.00			839	1.00			500	1.00		
Living alone	446	1.19	(0.68–2.05)		242	1.26	(0.61–2.61)		204	0.91	(0.38–2.17)		105	1.00	(0.27–3.74)		341	1.26	(0.69–2.32)		185	1.55	(0.78–3.10)		180	0.73	(0.26–2.06)		81	1.04	(0.16–6.55)	
Survey phase																																
Phase 1	2,400	1.00			1,200	1.00			1,200	1.00			885	1.00			1,515	1.00			800	1.00			1,019	1.00			581	1.00		
Phase 2	2,141	**1.53**	**(1.12–2.09)**	^**^	1,087	1.38	(0.87–2.19)		1,054	**1.67**	**(1.10–2.52)**	^*^	789	**1.83**	**(1.09–3.08)**	^*^	1,352	**1.40**	**(0.95–2.06)**	+	709	1.38	(0.89–2.16)		918	**1.65**	**(1.01–2.68)**	^*^	514	2.02	(0.82–4.99)	
Phase 3	2,200	**1.38**	**(1.01–1.87)**	^*^	1,111	1.11	(0.70–1.77)		1,089	**1.63**	**(1.08–2.46)**	^*^	812	**1.83**	**(1.09–3.06)**	^*^	1,388	1.18	(0.80–1.74)		710	1.56	(1.01–2.42)		947	1.30	(0.80–2.13)		543	0.99	(0.38–2.55)	
Phase 4	2,149	0.92	(0.66–1.27)		1,096	0.85	(0.52–1.38)		1,053	0.97	(0.63–1.50)		787	1.10	(0.64–1.89)		1,362	0.83	(0.56–1.25)		681	1.11	(0.70–1.75)		933	0.87	(0.53–1.46)		535	0.40	(0.13–1.27)	
Phase 5	2,155	**1.47**	**(1.08–2.01)**	^*^	1,094	1.21	(0.76–1.93)		1,061	**1.71**	**(1.13–2.59)**	^*^	794	1.38	(0.81–2.35)		1,361	1.53	(1.04–2.23)		688	**1.46**	**(0.94–2.28)**	+	938	**1.70**	**(1.05–2.74)**	^*^	529	0.91	(0.34–2.45)	
Phase 6	2,085	1.12	(0.81–1.54)		1,046	0.98	(0.60–1.59)		1,039	1.23	(0.80–1.89)		773	1.27	(0.74–2.18)		1,312	1.05	(0.70–1.56)		653	**1.48**	**(0.94–2.32)**	+	915	0.86	(0.51–1.45)		517	0.80	(0.29–2.23)	
Phase 7	2,086	1.06	(0.77–1.47)		1,055	0.89	(0.54–1.44)		1,031	1.22	(0.79–1.88)		770	**1.58**	**(0.92–2.71)**	+	1,316	0.85	(0.57–1.28)		652	1.06	(0.67–1.69)		911	1.13	(0.68–1.87)		523	0.88	(0.32–2.43)	
Phase 8	2,066	1.07	(0.77–1.47)		1,046	1.05	(0.65–1.69)		1,020	1.07	(0.69–1.66)		763	1.32	(0.77–2.27)		1,303	0.95	(0.64–1.43)		646	1.09	(0.69–1.72)		913	1.12	(0.68–1.86)		507	0.86	(0.31–2.39)	
Phase 9	2,080	1.18	(0.85–1.62)		1,057	0.91	(0.56–1.48)		1,023	1.43	(0.93–2.19)		766	1.02	(0.58–1.78)		1,314	1.25	(0.85–1.85)		643	1.41	(0.90–2.21)		910	1.07	(0.65–1.79)		527	0.71	(0.25–2.00)	
Interaction term																																
Living alone^*^Phase 1		1.00				1.00				1.00				1.00				1.00				1.00				1.00				1.00		
Living alone^*^Phase 2		0.95	(0.49–1.83)			0.86	(0.36–2.08)			1.20	(0.43–3.39)			0.85	(0.17–4.30)			1.02	(0.49–2.13)			0.66	(0.27–1.58)			1.91	(0.60–6.08)			1.23	(0.13–11.77)	
Living alone^*^Phase 3		0.85	(0.44–1.66)			1.00	(0.41–2.41)			0.79	(0.28–2.28)			1.04	(0.22–4.85)			0.90	(0.43–1.90)			**0.30**	**(0.12–0.75)**	^*^		**2.99**	**(0.95–9.40)**	+		3.42	(0.38–30.63)	
Living alone^*^Phase 4		1.61	(0.83–3.12)			1.36	(0.56–3.30)			2.24	(0.79–6.32)			1.32	(0.27–6.41)			1.76	(0.84–3.71)			0.72	(0.30–1.74)			**5.50**	**(1.75–17.33)**	^**^		1.83	(0.13–25.68)	
Living alone^*^Phase 5		1.46	(0.77–2.77)			1.63	(0.69–3.84)			1.47	(0.53–4.03)			2.68	(0.60–11.94)			1.25	(0.60–2.56)			0.89	(0.38–2.09)			**2.77**	**(0.89–8.61)**	+		4.56	(0.50–41.87)	
Living alone^*^Phase 6		**1.89**	**(0.99–3.64)**	+		1.58	(0.65–3.82)			**2.72**	**(1.00–7.43)**	+		1.17	(0.24–5.68)			**2.13**	**(1.02–4.42)**	^*^		0.96	(0.41–2.26)			**5.65**	**(1.78–17.89)**	^**^		1.94	(0.18–21.46)	
Living alone^*^Phase 7		**1.88**	**(0.97–3.63)**	+		1.45	(0.59–3.54)			**3.06**	**(1.12–8.40)**	^*^		1.20	(0.25–5.68)			**2.34**	**(1.11–4.92)**	^*^		1.18	(0.49–2.82)			**4.59**	**(1.45–14.51)**	^**^		1.39	(0.13–15.01)	
Living alone^*^Phase 8		1.38	(0.71–2.69)			1.04	(0.42–2.54)			2.16	(0.77–6.05)			1.15	(0.24–5.62)			1.52	(0.72–3.23)			0.67	(0.27–1.66)			**4.32**	**(1.37–13.57)**	^*^		1.45	(0.13–15.79)	
Living alone^*^Phase 9		1.38	(0.71–2.69)			1.35	(0.55–3.28)			1.74	(0.62–4.89)			1.99	(0.41–9.52)			1.24	(0.59–2.59)			0.66	(0.27–1.60)			**3.69**	**(1.16–11.74)**	^*^		3.93	(0.39–39.46)	

CI, confidence interval; OR, odds ratio.

All OR were adjusted by gender, age, residential area (prefecture), working status, personal annual income, education, smoking status, alcohol consumption, walking time, comorbidities (none, 1, 2+), and regular vaccination. +, ^*^, and ^**^ indicate statistically significant results in 10%, 5%, and 1% significance levels, respectively.

## DISCUSSION

### Summary of findings

We set out to determine whether changes occurred in psychological distress levels during different phases of the COVID-19 pandemic in 2020–2021, and whether those changes were affected by living arrangements, by conducting this repeated longitudinal study among 2,400 ordinary Japanese citizens. Overall, psychological distress among ordinary citizens increased during the early phase of the pandemic but has improved since then. However, the changes differed depending on living arrangements. Compared to those living with others, individuals living alone showed higher psychological distress levels during middle phases. Age- and gender-stratified analyses showed that SPD was especially more likely to develop among middle-aged women who lived alone. In addition, men who live alone or who are in the younger age group showed higher SPD proportion throughout the survey. Effective countermeasures targeting those who live alone or are of a young age group are essential.

### Comparison with past findings

There is a growing number of studies on the impact of COVID-19 on mental health. Most are cross-sectional studies; however, towards the end of 2020, some longitudinal studies have also been published.^22^ Some major longitudinal studies include those in the United Kingdom,^23^^,^^24^ the United States,^25^ Italy,^26^ Germany,^27^ and Argentina.^28^ However, few of these have referred to living arrangements. Fancourt et al. examined the trajectory of changes in living arrangements, depression, and anxiety scores, in 36,520 British subjects between March 23 and August 9, 2020, and found that those living alone showed less improvement in these scores than in other household configurations.^23^ Our study showed consistent results with their investigation; however, their study did not mention differences by age. Living alone among the young and the older populations bear different situations in terms of life stages. This study revealed that, in particular, those who are in the middle-aged groups who live alone are in need of more attention because they were more likely to continue being in a severe psychologically distressed state.

### Possible mechanism

In the early phase of the pandemic, psychological distress increased in both groups regardless of living arrangements. The rapid spread of COVID-19 created fear and anxiety about contracting the unknown virus,^29^^,^^30^ which may have caused increased distress of the whole population. After the early phase, however, distress was alleviated among those who live with others but did not change among those living alone. Although the number of infected people per day was decreasing, the government continued to restrict social contact, limiting opportunities for face-to-face contact. This restriction was continued throughout the year in Japan, so those living alone could not receive adequate social support and were more likely to feel severe loneliness.^10^^,^^31^^,^^32^ Loneliness is defined as “existence of a gap between the amount of social interaction desired and the amount of interaction possible,”^33^ and this can lead to serious problems, such as suicide, abuse, and cognitive decline.^7^^,^^8^ A recent cross-sectional study including 25,482 Japanese adults showed that the level of loneliness elevated after the COVID-19 pandemic.^34^ In order to avoid or alleviate such serious problems, it is necessary to implement mental health measures for people living alone. For example, encouraging online communication would be beneficial to prevent depression.^35^ In addition, it would be beneficial for alleviating loneliness to provide psychological therapies, such as mindfulness, lessons on friendship, robotic pets, and social facilitation software online.^36^

This study also showed that the association between living alone and deterioration of mental health was more apparent in the middle-aged group. In order to reduce face-to-face contact, the government recommends remote working, or online social events.^37^ However, previous studies have indicated that it is difficult for the middle-aged to older population to obtain sufficient social support through telephone or online communication.^38^^,^^39^ On the other hand, young people, even those who live alone, may be more likely to receive sufficient social support outside the family through online interactions. Furthermore, the study also found that mental health deterioration during the COVID-19 pandemic was more likely to have occurred in women living alone around September to October 2020. A past study showed that women’s psychological well-being is more dependent on the amount of social support than men.^40^ The negative impact given by deprivation of social support on mental health may be greater for women, especially for those living alone. The national statistics showed an excess suicide death rate between August and December 2020, when women showed higher likelihood of SPD in this study, suggesting the need for effective suicide prevention efforts. For example, internet-based cognitive behavioural therapy would be one choice because a recent randomized-controlled trial showed its’ effectiveness for reducing depressive mood during the COVID-19 pandemic.^41^

### Strengths and limitations

A major strength of this study is the multi-wave longitudinal study design, which consists of nine repeated measurements at regular intervals. In addition, the baseline survey was conducted just before the COVID-19 pandemic, allowing for accurate comparison between the pre- and post-pandemic periods. According to a recent systematic review, most of the longitudinal studies on COVID-19 pandemic and mental health started their investigation after the declaration of the pandemic by WHO.^42^ These studies cannot accurately assess the magnitude of the impact of the pandemic on mental health deterioration. However, there are some limitations in our study that should be considered. First, since the participants were recruited from among those who had enrolled at a single Internet research company, the results may have been affected due to selection bias. Those registered with the internet-survey company might be more likely to use the internet heavily. Heavy internet use is related to poor mental health; therefore, the study may include those with poorer mental health than population average.^36^ According to the Japanese Ministry of Internal Affairs and Communication, regular internet users were younger in age compared to non-users,^43^ so the results in this study may not be representative of the older population. Second, since the study participants were recruited from the Tokyo Metropolitan area, and not from all regions across Japan, the results may not be directly applicable to the general Japanese population. Compared to national statistics, this study shows lower proportion of older adults (24.9% of participants in this study and 28.8% of Japan’s entire population) and those who live alone (19.6% and 28.7%, respectively). Third, this study did not consider possible changes in independent variables, such as change of living arrangement or decline of personal income during follow-up. Fourth, no data on current or past history of medication for mental health were obtained for this study. If a certain number of participants had started medication during the period of these two surveys, the results may be biased. Lastly, living arrangements have shown to be associated with mental health, including not only whether one lives alone, but also with whom one lives.^44^^,^^45^ Due to the limited number of study participants, this study could not examine the relationship between living arrangement and mental health in detail. Future studies to examine the relationship using a larger data set is necessary.

### Conclusion

During the COVID-19 pandemic, those living alone are persistently at a higher risk of SPD compared to those living with others. Effective countermeasures targeting those living alone, such as enhancing online communication or providing psychological therapies, are essential.

## References

[1] World Health Organization. Coronavirus disease (COVID-2019) situation reports. Published 2020. Accessed June 20, 2020. https://www.who.int/emergencies/diseases/novel-coronavirus-2019/situation-reports.

[2] Galea S, Merchant RM, Lurie N. The Mental health consequences of COVID-19 and physical distancing: the need for prevention and early intervention. JAMA Intern Med. 2020;180(6):817–818. 10.1001/jamainternmed.2020.1562 32275292

[3] Blustein DL, Duffy R, Ferreira JA, Cohen-Scali V, Cinamon RG, Allan BA. Unemployment in the time of COVID-19: a research agenda. J Vocat Behav. 2020;119:103436. 10.1016/j.jvb.2020.103436 32390656 PMC7206417

[4] Gunnell D, Appleby L, Arensman E, . Suicide risk and prevention during the COVID-19 pandemic. Lancet Psychiatry. 2020;7(6):468–471. 10.1016/S2215-0366(20)30171-1 32330430 PMC7173821

[5] Nomura S, Kawashima T, Yoneoka D, . Trends in suicide in Japan by gender during the COVID-19 pandemic, up to September 2020. Psychiatry Res. 2021;295:113622. 10.1016/j.psychres.2020.113622 33290942

[6] World Health Organization. Policy brief: COVID-19 and the need for action on mental health. Published 2020. Accessed May 30, 2020. https://www.un.org/sites/un2.un.org/files/un_policy_brief-covid_and_mental_health_final.pdf.

[7] Grossman ES, Hoffman YSG, Palgi Y, Shrira A. COVID-19 related loneliness and sleep problems in older adults: worries and resilience as potential moderators. Pers Individ Dif. 2021;168:110371. 10.1016/j.paid.2020.110371 32904342 PMC7455172

[8] Palgi Y, Shrira A, Ring L, . The loneliness pandemic: Loneliness and other concomitants of depression, anxiety and their comorbidity during the COVID-19 outbreak. J Affect Disord. 2020;275:109–111. 10.1016/j.jad.2020.06.036 32658811 PMC7330569

[9] Fukushima N, Amagasa S, Kikuchi H, . Associations of older adults’ excursions from home with health-related physical activity and sedentary behavior. Arch Gerontol Geriatr. 2021;92:104276. 10.1016/j.archger.2020.104276 33069112

[10] Luchetti M, Lee JH, Aschwanden D, . The trajectory of loneliness in response to COVID-19. Am Psychol. 2020;75(7):897–908. 10.1037/amp0000690 32567879 PMC7890217

[11] Bu F, Steptoe A, Fancourt D. Who is lonely in lockdown? Cross-cohort analyses of predictors of loneliness before and during the COVID-19 pandemic. Public Health. 2020;186:31–34. 10.1016/j.puhe.2020.06.036 32768621 PMC7405905

[12] Kikuchi H, Machida M, Nakamura I, . Changes in psychological distress during the COVID-19 pandemic in Japan: a longitudinal study. J Epidemiol. 2020;30(11):522–528. 10.2188/jea.JE20200271 32963212 PMC7557175

[13] Tanaka T, Okamoto S. Increase in suicide following an initial decline during the COVID-19 pandemic in Japan. Nat Hum Behav. 2021;5(2):229–238. 10.1038/s41562-020-01042-z 33452498

[14] Machida M, Nakamura I, Saito R, . Adoption of personal protective measures by ordinary citizens during the COVID-19 outbreak in Japan. Int J Infect Dis. 2020;94:139–144. 10.1016/j.ijid.2020.04.014 32283285 PMC7194542

[15] Prime Minister of Japan and His Cabinet. Declaration of a state of emergency in response to the novel coronavirus disease (April 7). Published 2020. https://japan.kantei.go.jp/ongoingtopics/_00018.html.

[16] The Ministry of Health Labour and Welfare. Basic policies for novel coronavirus disease control by the government of Japan. Published online 2020. https://www.mhlw.go.jp/content/10900000/000617686.pdf.

[17] Kessler RC, Barker PR, Colpe LJ, . Screening for serious mental illness in the general population. Arch Gen Psychiatry. 2003;60(2):184–189. 10.1001/archpsyc.60.2.184 12578436

[18] Pratt LA. Serious psychological distress, as measured by the K6, and mortality. Ann Epidemiol. 2009;19(3):202–209. 10.1016/j.annepidem.2008.12.005 19217003

[19] Furukawa TA, Kawakami N, Saitoh M, . The performance of the Japanese version of the K6 and K10 in the World Mental Health Survey Japan. Int J Methods Psychiatr Res. 2008;17(3):152–158. 10.1002/mpr.257 18763695 PMC6878390

[20] Furukawa TA, Kessler RC, Slade T, Andrews G. The performance of the K6 and K10 screening scales for psychological distress in the Australian National Survey of Mental Health and Well-Being. Psychol Med. 2003;33(2):357–362. 10.1017/S0033291702006700 12622315

[21] Hedeker D, Gibbons RD. A random-effects ordinal regression model for multilevel analysis. Biometrics. 1994;50(4):933–944. 10.2307/2533433 7787006

[22] Vindegaard N, Benros ME. COVID-19 pandemic and mental health consequences: systematic review of the current evidence. Brain Behav Immun. 2020;89:531–542. 10.1016/j.bbi.2020.05.048 32485289 PMC7260522

[23] Fancourt D, Steptoe A, Bu F. Trajectories of anxiety and depressive symptoms during enforced isolation due to COVID-19 in England: a longitudinal observational study. Lancet Psychiatry. 2021;8(2):141–149. 10.1016/S2215-0366(20)30482-X 33308420 PMC7820109

[24] Chandola T, Kumari M, Booker CL, Benzeval MJ. The mental health impact of COVID-19 and pandemic related stressors among adults in the UK. medRxiv. Published online 2020. doi:10.1101/2020.07.05.20146738. 10.1101/2020.07.05.20146738 PMC7783135 33280639

[25] Holman EA, Thompson RR, Garfin DR, Silver RC. The unfolding COVID-19 pandemic: a probability-based, nationally representative study of mental health in the United States. Sci Adv. 2020;6(42):eabd5390. 10.1126/sciadv.abd5390 32948511 PMC7556755

[26] Fiorillo A, Sampogna G, Giallonardo V, . Effects of the lockdown on the mental health of the general population during the COVID-19 pandemic in Italy: results from the COMET collaborative network. Eur Psychiatry. 2020;63(1):e87. 10.1192/j.eurpsy.2020.89 32981568 PMC7556907

[27] Zacher H, Rudolph CW. Individual differences and changes in subjective wellbeing during the early stages of the COVID-19 pandemic. Am Psychol. 2021;76(1):50–62. 10.1037/amp0000702 32700938

[28] Canet-Juric L, Andrés ML, Del Valle M, . A longitudinal study on the emotional impact cause by the COVID-19 pandemic quarantine on general population. Front Psychol. 2020;11(September):565688. 10.3389/fpsyg.2020.565688 33071893 PMC7531077

[29] Brooks SK, Webster RK, Smith LE, . The psychological impact of quarantine and how to reduce it: rapid review of the evidence. Lancet. 2020;395(10227):912–920. 10.1016/S0140-6736(20)30460-8 32112714 PMC7158942

[30] Bao Y, Sun Y, Meng S, Shi J, Lu L. 2019-nCoV epidemic: address mental health care to empower society. Lancet. 2020;395(10224):e37–e38. 10.1016/S0140-6736(20)30309-3 32043982 PMC7133594

[31] Banerjee D, Rai M. Social isolation in Covid-19: the impact of loneliness. Int J Soc Psychiatry. 2020;66(6):525–527. 10.1177/0020764020922269 32349580 PMC7405628

[32] Killgore WDS, Cloonan SA, Taylor EC, Dailey NS. Loneliness: a signature mental health concern in the era of COVID-19. Psychiatry Res. 2020;290:113117. 10.1016/j.psychres.2020.113117 32480121 PMC7255345

[33] Jeste DV, Lee EE, Cacioppo S. Battling the modern behavioral epidemic of loneliness. JAMA Psychiatry. 2020;77(6):553–554. 10.1001/jamapsychiatry.2020.0027 32129811 PMC7483387

[34] Murayama H, Okubo R, Tabuchi T. Increase in social isolation during the COVID-19 pandemic and its association with mental health: findings from the JACSIS 2020 Study. Int J Environ Res Public Heal. 2021;18(16):8238. 10.3390/ijerph18168238 34443988 PMC8394951

[35] Nakagomi A, Shiba K, Kondo K, Kawachi I. Can online communication prevent depression among older people? A Longitudinal Analysis. J Appl Gerontol. 2022;41(1):167–175. 10.1177/0733464820982147 33356760

[36] Spada MM. An overview of problematic Internet use. Addict Behav. 2014;39(1):3–6. 10.1016/j.addbeh.2013.09.007 24126206

[37] The Ministry of Health Labour and Welfare in Japan. 10 tips for reducing contact. Published 2020. Accessed February 7, 2021. https://www.mhlw.go.jp/content/10900000/000628616.pdf.

[38] Xie B, Charness N, Fingerman K, Kaye J, Kim MT, Khurshid A. When going digital becomes a necessity: ensuring older adults’ needs for information, services, and social inclusion during COVID-19. J Aging Soc Policy. 2020;32(4–5):460–470. 10.1080/08959420.2020.1771237 32507061 PMC8855980

[39] Teo AR, Choi H, Andrea SB, . Does mode of contact with different types of social relationships predict depression in older adults? Evidence from a nationally representative survey. J Am Geriatr Soc. 2015;63(10):2014–2022. 10.1111/jgs.13667 26437566 PMC5527991

[40] Hori M, Kamo Y. Gender differences in happiness: the effects of marriage, social roles, and social support in East Asia. Appl Res Qual Life. 2018;13(4):839–857. 10.1007/s11482-017-9559-y

[41] Oehler C, Scholze K, Reich H, Sander C, Hegerl U. Intervention use and symptom change with unguided internet-based cognitive behavioral therapy for depression during the COVID-19 pandemic: log data analysis of a convenience sample. JMIR Ment Health. 2021;8(7):e28321. 10.2196/28321 34115604 PMC8288646

[42] Richter D, Riedel-Heller S, Zürcher SJ. Mental health problems in the general population during and after the first lockdown phase due to the SARS-Cov-2 pandemic: rapid review of multi-wave studies. Epidemiol Psychiatr Sci. 2021;30:e27. 10.1017/S2045796021000160 33685551 PMC7985862

[43] Ministry of Internal Affairs and Communication of Japan (MIC). *White Paper on Information and Communications in Japan* *2019*; 2019. Accessed July 11, 2020. https://www.soumu.go.jp/johotsusintokei/whitepaper/eng/WP2019/2019-index.html.

[44] Kikuchi H, Takamiya T, Odagiri Y, . Gender differences in association between psychological distress and detailed living arrangements among Japanese older adults, aged 65–74 years. Soc Psychiatry Psychiatr Epidemiol. 2014;49(5):823–830. 10.1007/s00127-013-0778-8 24126557

[45] Honjo K, Tani Y, Saito M, . Living alone or with others and depressive symptoms, and effect modification by residential social cohesion among older adults in Japan: the JAGES longitudinal study. J Epidemiol. 2018;28(7):315–322. 10.2188/JEA.JE20170065 29398683 PMC6004365

